# Improving the break space within in a liaison psychiatry setting – a pathway to staff wellbeing and better teamwork?

**DOI:** 10.1192/j.eurpsy.2026.11964

**Published:** 2026-07-31

**Authors:** A. Prabhu, R. Hotchkin, I. Tarbet, C. C. Sin Fai Lam

**Affiliations:** King’s College Hospital, London, United Kingdom

## Abstract

**Introduction:**

Having adequate breaks is recognised as a vital contributor to staff wellbeing at work and productivity. This is increasingly relevant with rising levels of burnout and poor workplace satisfaction. A key component of adequate rest is the existence of a suitable ‘third’ space. Studies of design features of break spaces for healthcare professionals have highlighted the importance of proximity to workstation, provision of plants/outdoor areas and opportunities for socialisation to build professional relationships with colleagues.

**Objectives:**

We undertook a needs analysis and found that while the liaison psychiatry team had a break space in close proximity to their workstation, it was not regularly used as it was not felt to be a welcoming or practical space. This was identified as a potential risk factor for fragmentation and worsening mental health within the team. We therefore undertook to improve the break space to increase staff wellbeing and team cohesiveness.

**Methods:**

We performed two PDSA cycles informed by qualitative surveys of staff members.

**Results:**

Our initial survey showed that only 43% of staff used the break area and 15% of staff found it pleasant to use. 29% of staff found it was a relaxing area, said it contributed to their wellbeing at work, and felt refreshed after using the break area. Only 43% said the area enabled them to build relationships with the MDT. 15% found that facilities were appropriate for their needs. As our first intervention, we updated the break space with the inclusion of a reading area. This aimed to provide opportunities for education, leisure and to encourage topics of conversation. We also included indoor plants, ornaments and art to provide a calming environment. Post this, use of the area increased from 57% to 75%. 75% found it pleasant to use compared to 15% previously. 75% of people now found the area relaxing to use. 100 % of people felt refreshed after using it and felt it contributed to their wellbeing at work. 50% felt the break area now had appropriate facilities and 75% felt the break area enabled them to build relationships with other members of the MDT. Our second intervention focused on improving the comfort of the area with the addition of comfortable blankets and pillows. Post the second intervention, the percentage of those using the area remained at 75%. However, 100% now found it pleasant to use, felt refreshed after their breaks, felt the break area contributed to their wellbeing at work, found the area had appropriate facilities, and enabled them to build relationships with other members of the MDT.

**Conclusions:**

This quality improvement project demonstrates how low-cost interventions to improve staff break areas can have a significant impact on staff wellbeing and staff attitudes to networking with the MDT. This may have future effects on productivity of staff, teamworking and retention of staff.

**Disclosure of Interest:**

None Declared

